# Impact of additional module training on the level of basic life support knowledge of first year students at the University of Maribor

**DOI:** 10.1186/1865-1380-4-16

**Published:** 2011-04-19

**Authors:** Damjan Lešnik, Bojan Lešnik, Jerneja Golub, Miljenko Križmarić, Štefan Mally, Štefek Grmec

**Affiliations:** 1 Center for Emergency Medicine, Ulica talcev 9, 2000 Maribor, Slovenia; 2 Faculty of Medicine, University of Maribor, Slomškov trg 15, 2000 Maribor, Slovenia; 3 Faculty of Health Sciences,Žitna ulica 15, 2000 Maribor; Slovenia; 4 Center for Emergency Medicine, Ulica talcev 9, 2000 Maribor, Slovenia; 5 Faculty of Medicine, University of Ljubljana, Vrazov trg 2,1104 Ljubljana, Slovenia

## Abstract

**Aim:**

The aim of this study was to investigate the impact of additional (two versus one session) basic life support (BLS) training of university students on knowledge and attitude concerning the performance of cardiopulmonary resuscitation.

**Methods:**

A total of 439 students in three separate groups were tested: those with no prior BLS training; BLS training in high school (part of the driver's education course); and BLS training in high school (in the driver's education course) and additional BLS training at the university.

**Results:**

Our study showed the best results of BLS education in a group of university students who took an additional BLS module approximately half a year after the driver's education BLS course. In our study we observed equal levels of knowledge between the group with BLS training in high school and the group without any formal BLS education. The questionnaire revealed a disappointing level of knowledge about BLS in both groups.

**Conclusion:**

Additional basic life support training (two BLS training sessions: high school and university) improves retention of knowledge and attitudes concerning performing CPR in first year university students.

## Introduction

Recent studies have emphasized that bystander cardiopulmonary resuscitation (CPR) is a very important contributing factor in the survival of out-of-hospital cardiac arrest (OHCA) patients [1-10]. The practice of basic life support (BLS) by lay people is therefore essential for sufficient functioning of the chain of survival and is definitely an important part of effective emergency services for a patient needing resuscitation [11]. However, lay people can only play their role within the chain of survival if they are adequately trained and if continuous repetitions of relevant training information are offered and used [12]. Introducing CPR training in high school and university settings has been widely recommended as a long-term strategy to educate the wider community. In general, students have poor theoretical knowledge, although most of them are willing and motivated to learn CPR [12-17]. A pyramidal teaching approach involving students who had BLS training shows potential for spreading BLS knowledge to lay people [18]. In a previous study we confirmed that the potential bystander in our community is generally poorly educated about performing CPR, but willing to gain knowledge and skills in BLS and to follow dispatchers' instructions [19].

The aim of this study was to investigate the impact of additional basic life support (BLS) training of university students on knowledge and attitude for performing cardiopulmonary resuscitation (one training session vs. two training sessions).

## Methods

The study was conducted in the context of the campaign program "Education of lay people in BLS in the Maribor area" and arranged by the authors. The campaign program was designed to facilitate the wider dissemination of BLS skills and knowledge in the local population.

Data for the study were collected in the spring of 2009. We compared the knowledge of BLS in three university student groups at the end of the first year of college: those with no prior BLS training, those who had BLS training in high school (in the driver's education course) approximately 13 months before testing, and those who had BLS training in high school and additional BLS training at the university (within 6 months after the driver's education BLS course and approximately 8 months before testing). Approximately 90% of high school students successfully accomplished the driver's education course. The 10% of high school students who failed the driver's education course (and thus did not have any other opportunity for BLS training in high school) would therefore be expected to perform poorly in the BLS program at the university level. Participants in the first two groups were students of the Faculty of Electrical Engineering/Computer Science and of the Faculty of Education. In the third group were students of the Faculty of Medicine and Faculty of Health Sciences.

The driver's education BLS courses were guided by health workers who are first aid instructors certified by the Slovenian Red Cross organization and supervised by the Slovenian Resuscitation Council (pyramid teaching methods). The driver's education BLS training is part of the first aid course for all driver's license candidates. The BLS training includes a lecture (1 h) and practical training in small groups (4 h) in accordance with European Resuscitation Council (ERC) recommendations. The additional BLS module was given by ERC instructors. The driver's education BLS training and the BLS module have identical structures, presentations, contents, tools and equipment. Both groups were trained by ERC instructors. The Red Cross group had ERC licenses for the BLS course, and the ERC group had licenses for the BLS and Advanced Life Support course.

Knowledge of BLS was tested with a questionnaire in accordance with the 2005 ERC guidelines for BLS and approved by the deans of all four faculties, who were informed about the results at the end of the study and were advised of a concrete plan of action to improve the BLS knowledge of their students. According to the Declaration of Helsinki, data were made anonymous [20]. A standardized questionnaire with 28 items (see Tables 1, 2 and 3) included checkboxes or open answer areas, and contained information about the intention of the survey. It was presented to students and collected personally by the authors at the end of testing in hard form.

**Table 1:** Comparison between the group with a driver's education BLS course followed by BLS module training and the group with the driver's education BLS course only

Question (correct answer)	BLS driver's education course + BLS module course	BLS driver's education course only	p-value
	N = 197	N = 179	
Gender [Male/all]	Male: 45/197 (23%)	Male: 65/179 (36%)	0.004

Age [years ± SD]	19.6 ± 0.7 (18-22)	19.7 ± 0.9 (19-24)	NS

Are you living in an urban area?	Yes: 69/197 (35%)	Yes: 57/179 (32%)	NS

Would you recognize a situation in which CPR is needed? §	Mean ± SD: 4.4 ± 0.6	Mean ± SD: 3.9 ± 0.7	<0.001

In your opinion, how well do you know BLS? §§	Mean ± SD: 3.6 ± 0.6	Mean ± SD: 2.9 ± 0.6	<0.001

In your opinion, how effectively would you perform CPR? §§	Mean ± SD: 3.4 ± 0.5	Mean ± SD: 2.8 ± 0.6	<0.001

Would you perform rescue breathing in everyone when needed?	Yes: 197/197 (100%)	Yes: 172/179 (96%)	0.005

What is the emergency telephone number? (112)	Correct answer: 196/197 (99%)	Correct answer: 157/179 (88%)	<0.001

How long should a lay person perform CPR? (until the rescue unit arrives)	Correct answer: 89/197 (45%)	Correct answer: 28/179 (16%)	<0.001

Have you ever done CPR?	Yes: 20/197 (10%)	Yes: 13/179 (7%)	0.323

Have you ever seen someone to loose consciousness?	Yes: 74/197 (38%)	Yes: 68/179 (38%)	0.932

Would you be willing to listen to dispatcher's instructions and perform CPR untill the rescue unit arrives?	Yes: 107/197 (54%)	Yes: 119/179 (67%)	0.016

Would you do CPR with no scruples about it, when necessary?	Yes: 84/197 (43%)	Yes: 28/179 (16%)	<0.001

Did you get the most of your knowledge of CPR at BLS driver course?	Yes: 105/197 (53%)	Yes: 160/179 (89%)	<0.001

Are you interested in gaining more skills and knowlege of BLS?	Yes: 191/197 (97%)	Yes: 165/179 (92%)	0.039

Would you be willing to gain additional BLS knowlege in a form of BLS courses?	Yes: 154/197 (78%)	Yes: 107/179 (60%)	<0.001

What is the correct position of hands when performing compressions? (on sternum in the middle of the chest)	Correct answer: 186/197 (94%)	Correct answer: 139/179 (78%)	<0.001

What is CPR ratio between chest compressions and breathing in adults? (30:2)	Correct answer: 189/197 (189%)	Correct answer: 70/179 (39%)	<0.001

Frequency of chest compressions in adults? (100/min)	Correct answer: 157/197 (80%)	Correct answer: 33/179 (18%)	<0.001

The depth of chest compressions for effective CPR is? (4-5 cm)	Correct answer: 150/197 (76%)	Correct answer: 61/179 (34%)	<0.001

What is Heimlich sign? (grasping one`s own throat unable to breath)	Correct answer: 151/197 (77%)	Correct answer: 51/179 (29%)	<0.001

How would you do rescue breathing in mouth to mouth resuscitation? (blow steadily about 1s as in normal breathing)	Correct answer: 83/197 (42%)	Correct answer: 21/179 (12%)	<0.001

What is important for effective CPR in adults? (to do chest compessions together with rescue breathing)	Correct answer: 140/197 (71%)	Correct answer: 143/179 (80%)	0.048

What is the purpose of automatic defibrillator (AED)? (to end some of lifethreating heart rhythm disturbances)	Correct answer: 129/197 (66%)	Correct answer: 79/179 (44%)	<0.001

What is the sequence of CPR when using AED? (CPR is performed as usual, additionally placing the electrodes and following the AED instruction)	Correct answer: 83/197 (42%)	Correct answer: 64/179 (36%)	0.206

§ Five-point Likert scale: 5- always, 1- never.

§§ Five-point Likert scale: 5-excellent, 1-extremely poor.

**Table 2:** Comparison between the group with no BLS training and the group with the driver's education BLS course only

Question (correct answer)	No BLS training	BLS driver's education course only	p-value
	N = 63	N = 179	
Gender [Male/Female]	Male: 9/63 (14%)	Male: 65/179 (36%)	<0.001

Age [years ± SD]	19.1 ± 0.8 (18-21)	19.7 ± 0.9 (19-24)	<0.001

Are you living in an urban area?	Yes: 19/63 (30%)	Yes: 57/179 (32%)	0.804

Would you recognize a situation in which CPR is needed? §	Mean ± SD: 3.5 ± 0.5	Mean ± SD: 3.9 ± 0.7	<0.001

In your opinion, how well do you know BLS? §§	Mean ± SD: 3.6 ± 0.6	Mean ± SD: 2.9 ± 0.6	<0.001

In your opinion, how effectively would you perform CPR? §§	Mean ± SD: 2.6 ± 0.6	Mean ± SD: 2.8 ± 0.6	0.024

Would you perform rescue breathing in everyone when needed ?	Yes: 46/63 (73%)	Yes: 172/179 (96%)	<0.001

What is the emergency telephone number? (112)	Yes: 58/63 (92%)	Yes: 157/179 (88%)	0.345

How long should a lay person perform CPR? (until the rescue unit arrives)	Correct answer: 8/63 (13%)	Correct answer: 28/179 (16%)	0.572

Have you ever done CPR?	Yes: 2/63 (3%)	Yes: 13/179 (7%)	0.247

Have you ever seen someone to loose consciousness?	Yes: 22/63 (35%)	Yes: 68/179 (38%)	0.665

Would you be willing to listen to dispatcher's instructions and perform CPR untill the rescue unit arrives?	Yes: 58/63 (92%)	Yes: 119/179 (67%)	<0.001

Would you do CPR with no scruples about it, when necessary?	Yes: 7/63 (11%)	Yes: 28/179 (16%)	0.3792

Are you interested in gaining more skills and knowlege of BLS?	Yes: 63/63 (100%)	Yes: 165/179 (92%)	0.0222

Would you be willing to gain additional BLS knowlege in a form of BLS courses?	Yes: 61/63 (97%)	Yes: 107/179 (60%)	<0.001

What is the correct position of hands when performing compressions? (on sternum in the middle of the chest)	Correct answer: 48/63 (76%)	Correct answer: 139/179 (78%)	0.812

What is CPR ratio between chest compressions and breathing in adults? (30:2)	Correct answer: 23/63 (37%)	Correct answer: 70/179 (39%)	0.715

Frequency of chest compressions in adults? (100/min)	Correct answer: 10/63 (16%)	Correct answer: 33/179 (18%)	0.647

The depth of chest compressions for effective CPR is? (4-5 cm)	Correct answer: 21/63 (33%)	Correct answer: 61/179 (34%)	0.914

What is Heimlich sign? (grasping one`s own throat unable to breath)	Correct answer: 18/63 (29%)	Correct answer: 51/179 (29%)	0.990

How would you do rescue breathing in mouth to mouth resuscitation? (blow steadily about 1s as in normal breathing)	Correct answer: 8/63 (13%)	Correct answer: 21/179 (12%)	0.839

What is important for effective CPR in adults? (to do chest compessions together with rescue breathing)	Correct answer: 49/63 (78%)	Correct answer: 143/179 (80%)	0.722

What is the purpose of automatic defibrillator (AED)? (to end some of lifethreating heart rhythm disturbances)	Correct answer: 27/63 (43%)	Correct answer: 79/179 (44%)	0.861

What is the sequence of CPR when using AED? (CPR is performed as usual, additionally placing the electrodes and following the AED instruction)	Correct answer: 21/63 (33%)	Correct answer: 64/179 (36%)	0.729

§ Five-point Likert scale: 5- always, 1- never.

§§ Five-point Likert scale: 5-excellent, 1-extremely poor.

**Table 3:** Comparison between the group of the Faculty of Health Sciences and the group of the Faculty of Medicine

Question (correct answer)	Faculty of Health Sciences	Faculty of Medicine	p-value
	**N = 97**	**N = 100**	

Gender [Male/Female]	Male: 10/97 (10%)	Male: 35/100 (35%)	<0.001

Age [years ± SD]	19.7 ± 0.7 (19-22)	19.5 ± 0.7 (18-22)	0.914

Are you living in an urban area?	Yes: 28/97 (29%)	Yes: 41/100 (41%)	0.074

Would you recognize a situation in which CPR is needed? §	Mean ± SD: 4.4 ± 0.5	Mean ± SD: 4.4 ± 0.6	0.685

In your opinion, how well do you know BLS? §§	Mean ± SD: 3.6 ± 0.6	Mean ± SD: 3.6 ± 0.6	0.928

In your opinion, how effectively would you perform CPR? §§	Mean ± SD: 3.4 ± 0.5	Mean ± SD: 3.4 ± 0.6	0.864

Would you perform rescue breathing in everyone when needed ?	Yes: 97/97 (100%)	Yes: 100/100 (100%)	-

What is the emergency telephone number? (112)	Yes: 96/97 (99%)	Yes: 100/100 (100%)	0.309

How long should a lay person perform CPR? (until the rescue unit arrives)	Correct answer: 43/97 (44%)	Correct answer: 46/100 (46%)	0.814

Have you ever done CPR?	Yes: 11/97 (11%)	Yes: 9/100 (9%)	0.587

Have you ever seen someone to loose consciousness?	Yes: 42/97 (43%)	Yes: 32/100 (32%)	0.102

Would you be willing to listen to dispatcher's instructions and perform CPR untill the rescue unit arrives?	Yes: 66/97 (68%)	Yes: 41/100 (41%)	<0.001

Would you do CPR with no scruples about it, when necessary?	Yes: 38/97 (39%)	Yes: 46/100 (46%)	0.333

Did you get the most of your knowledge of CPR at BLS driver course?	Yes: 54/97 (56%)	Yes: 51/100 (51%)	0.511

Are you interested in gaining more skills and knowlege of BLS?	Yes: 94/97 (97%)	Yes: 97/100 (97%)	0.970

Would you be willing to gain additional BLS knowlege in a form of BLS courses?	Yes: 79/97 (81%)	Yes: 75/100 (75%)	0.274

What is the correct position of hands when performing compressions? (on sternum in the middle of the chest)	Correct answer: 90/97 (93%)	Correct answer: 96/100 (96%)	0,326

What is CPR ratio between chest compressions and breathing in adults? (30:2)	Correct answer: 94/97 (97%)	Correct answer: 95/100 (95%)	0.498

Frequency of chest compressions in adults? (100/min)	Correct answer: 77/97 (79%)	Correct answer: 80/100 (80%)	0.914

The depth of chest compressions for effective CPR is? (4-5 cm)	Correct answer: 76/97 (78%)	Correct answer: 74/100 (74%)	0.474

What is Heimlich sign? (grasping one`s own throat unable to breath)	Correct answer: 83/97 (86%)	Correct answer: 68/100 (68%)	0.004

How would you do rescue breathing in mouth to mouth resuscitation? (blow steadily about 1s as in normal breathing)	Correct answer: 33/97 (34%)	Correct answer: 50/100 (50%)	0.023

What is important for effective CPR in adults? (to do chest compessions together with rescue breathing)	Correct answer: 61/97 (63%)	Correct answer: 79/100 (79%)	0.013

What is the purpose of automatic defibrillator (AED)? (to end some of lifethreating heart rhythm disturbances)	Correct answer: 47/97 (48%)	Correct answer: 82/100 (82%)	<0.001

What is the sequence of CPR when using AED? (CPR is performed as usual, additionally placing the electrodes and following the AED instruction)	Correct answer: 39/97 (40%)	Correct answer: 44/100 (44%)	0.590

§ Five-point Likert scale: 5- always, 1- never.

§§ Five-point Likert scale: 5-excellent, 1-extremely poor.

Comparisons were made among the groups and within the third group with the BLS module course, where the collected data were compared between the two faculties (Faculty of Medicine and Faculty of Health Sciences). Statistical analyses were performed using SPSS for Windows, release 12.0; SPSS, Chicago IL. Wilcoxon signed rank test, t-test and Fisher's exact test were used where appropriate. Descriptive values of variables were expressed as average, standard deviation and percentages. Power analysis was made by using G-Power™ 3.0.10 for Microsoft Windows XP™ (Microsoft Inc., Redmond, WA). Wilcoxon signed rank test, t-test and Fisher's exact test were used where appropriate. For data not normally distributed, the Wilcoxon signed rank test was used. All *p *values of less than 0.05 were considered to indicate statistical significance.

## Results

A total of 439 students (118 men and 321 women; average age 19.5 +/- 0.8 years) participated in the study. There were 197 participants with additional BLS training (Faculty of Medicine and Faculty of Health Sciences), 179 participants who had taken the driver's education BLS course and 63 participants who had had no BLS training. All results are shown in Tables 1, 2 and 3.

Compared to the group who received an additional BLS training module, we found that the group with BLS training from the driver's education course was more willing to follow dispatchers' instructions by telephone to perform CPR (67% vs. 54%; *p *< 0.05) and less willing to take BLS training, especially in course form (*p *< 0.001) (Table 1). Only 16 percent of students with BLS training from the driver's education course were prepared to start CPR without any delay when necessary compared to 43 percent in the group that had received the BLS module (*p *< 0.001). On the five-point Likert scale, we found higher results of self-assurance in one's own knowledge in the group with the BLS module (*p *< 0.001). The same group was better informed about the emergency number in Slovenia (*p *< 0.001). There was also a significantly higher rate of correct answers about closed chest compression, breathing/ventilation, recognizing the Heimlich sign, automatic defibrillator use and how to approach unconscious victims in the BLS module group (*p *< 0.001).

When comparing the groups with and without the driver's education BLS course (Table 2), we found no statistical differences in knowledge about closed chest compression, breathing/ventilation, the Heimlich sign, automatic defibrillator use and how to approach unconscious victims. The group without a BLS course was more willing to follow dispatchers' instructions by telephone to perform CPR (92% vs. 67%; *p *< 0.001) and to take part in a BLS training course (97% vs. 60%; *p *< 0.001). The same group showed less self-assurance in their own knowledge of BLS (measured by five-point Likert scale; *p *< 0.001).

Within the group with the BLS module, we compared the students of the Faculty of Medicine with students of the Faculty of Health Sciences. Medical students have better knowledge of ventilation (*p *= 0.02), automatic defibrillator use (*p *< 0.001) and coordination in CPR (*p *= 0.01). The students of the Faculty for Health Sciences were more willing to follow dispatchers' instructions by telephone (*p *< 0.001) and recognized the Heimlich sign better (*p *= 0.004).

## Discussion

Since previous studies have found that university students showed poor theoretical knowledge and demonstrated willingness and motivation for courses on BLS [12-17], we sought to examine characteristics of students of the University of Maribor. Our survey suggests that an additional module of BLS training in the first academic year improves theoretical knowledge in students and their preparedness to perform CPR. This study also highlights some notable differences between the two faculties inside the group of students who received an additional BLS module.

Perkins et al. [21] found that care of the acutely ill patient in the hospital is often suboptimal. Poor recognition of critical illness combined with a lack of knowledge and failure to appreciate clinical urgency of a situation has been identified as a contributory factor. They confirmed that the present training of medical students in these important skills is fragmented. The nominal group in this study identified 71 essential and 16 optional competencies that students should possess by graduation and proposed that these competencies should form a core set for undergraduate training in resuscitation and acute care [21]. Beckers et al. reported on the Medical Reform Curriculum Aachen, which has a 3-week interdisciplinary introduction to emergency medical care in the first semester of medical school. Besides skill training in the basics of emergency medical care (BLS, early defibrillation), practical training in other lifesaving techniques (e.g., immobilization skills) and basic principles of daily clinical care are included. The course evaluation data clearly showed acceptance of the new approach and enhanced possibilities of extending implementation of relevant topics concerning emergency medical care within the Medical Reform Curriculum Aachen [22]. Das and Elzubeir confirmed the importance of training physicians and other health care professionals in first aid and BLS in the form of formal training in the first year of medical school. In their study they found that students were uniformly enthusiastic and highly motivated by the program [23]. Self-assessed confidence in the ability to perform skills on their own after completing the program was moderately correlated with the perceived frequency of opportunity to practice many skills. There was nevertheless a consistent desire for more time to practice.

In the curriculum of first year students at the Faculty of Medicine and the Faculty of Health Sciences in Maribor, formal BLS training as a BLS module course and practical training in other lifesaving techniques have been incorporated. As we confirmed in our investigation, students with this form of BLS education have shown better theoretical knowledge and higher confidence in their own skills than students with BLS training from the driver's education course and have been more motivated for additional learning in the form of courses. As the results of our survey together with those of several other studies suggest, BLS education in a module form is very effective and could become obligatory for all university and high school students [14,15,17,24-27], or even for those in primary school [16,27,28]. Our agreements with deans of different faculties in Maribor are in the final phase, and we are close to starting a major campaign of BLS courses at our university [19,29]. In our survey, we observed a substantial portion of students who had insufficient knowledge and skills to perform effective ventilation in CPR after BLS training. This suggests that the courses in the first year of college should be oriented to effective chest compression, safe and early defibrillation, and good communication with the dispatcher.

We have demonstrated that there are no differences in key points of BLS knowledge between students with driver's education BLS training and students without BLS training. This observation takes into consideration the quality of driver's education BLS first aid courses organized by the national Red Cross. In our opinion reevaluation of the first aid instructor education and of the form of BLS training in driver's education courses is necessary. Our results confirm that retention of CPR skills is poor. The solution for this problem is to improve instructor training and to use contemporary teaching methods (more frequent refresher courses for instructors). Also a general testing of knowledge retention with uniform criteria should be applied [30,31]. Detailed CPR quality assessment, careful monitoring of the quality of instructions, awareness of instructors when inadequate CPR is demonstrated and feedback should be integrated into the training to ensure optimal performance in a real-life resuscitation - it is time to stop the blind leading the blind [32]. Optimal refresher training in BLS is another option for better retention of CPR skills. Refresher training intervals should not exceed 7 months [33,34]. Maybe our two-step method (driver's education BLS course followed by BLS module training) is a practical way to improve the retention of key BLS knowledge. Traditional classroom or simulation-based learning could be combined with DVD or website self-instructional systems in order to reach and reinstruct individuals who are unwilling to participate in a live course [35-37]. Medical students of the Faculty of Medicine have founded a society called "For Life." This group of young people, under the supervision of ERC instructors and teachers, organizes BLS and first aid courses for lay persons in various places. In 2009 they successfully carried out 44 BLS courses with 1,110 participants, including school children. Their next project is a campaign in primary schools, where more medical students will participate as instructors in BLS training [38].

## Limitations

Our study has some limitations. The group of students without BLS training was relatively small, so the results can only partially be transferred to a general population of students with no prior BLS training. We have compared two different levels of BLS trainers (BLS vs. BLS + ALS trainers), but both were instructed in accordance with the methodical and didactic instructions of the ERC as recommended in the 2005 ERC Guidelines. We compared BLS knowledge among three different faculties, and nurses/medical students probably had some interest and affinity compared to teachers and engineers. However, in the group with no prior BLS training we observed great willingness to gain knowledge and skills in BLS and to follow dispatchers' instructions. This information supports ideas about interest and motivation in non-medical students. The primary goal of this article is to motivate other faculty members to help with the progress in improving teaching methods in BLS training and reactivating BLS education for all university students.

## Conclusion

Our study has shown the best results for BLS education in a group of university students who took an additional BLS module approximately a half year after the driver's education BLS course. The two-step method is a practical way to improve the retention of knowledge concerning BLS. We did not observe any differences in theoretical knowledge between the group of students who had had BLS training during the driver's education course and the group without any formal BLS education. The questionnaire revealed a disappointing level of knowledge of the fundamentals of BLS in both groups. However, there was a welcomed willingness of these students as potential bystanders to take BLS training and to follow dispatchers' instructions by telephone if CPR is indicated. We must recognize this fact as an emergency call to organize BLS module courses for all university students in the first year of study.

## Competing interests

The authors declare that they have no competing interests.

## Authors' contributions

GŠ conceived of the study, and participated in its design and coordination. LD, LB and GJ participated in acquisition of data, LD and LB also contributed to concepcion of the study. KM participated in interpretation of data and performed the statistical analysis. MŠ participated in the design of the study and drafted the manuscript. All authors read and approved the final manuscript.

## Authors' information

**Štefek Grmec **has gathered clinical experience for the last 18 years, and focused his scientific and research interests on the areas of emergency medicine (in general), organization of emergency medical services, difficult intubation and airway management in the field, capnometry and capnography in CPR and shock, acute medicine and family practice, trauma in the prehospital setting, severe head injury in the prehospital setting, RSI, vasopressin, erythropoietin in CPR, echocardiography in the prehospital setting, cardiac biomarkers in heart failure and ultrasound. He has concluded postgraduate study (4 years) in emergency and intensive medicine. He is national coordinator of the residents' program and specialization of emergency medicine in Slovenia. His contributions have been published in the following journals: *Intensive Care Medicine, European Journal of Emergency Medicine, Critical Care, Emergency Medicine Journal, Academic Emergency Medicine, Prehospital and Disaster Medicine, Acta Anaesthesiologica Scandinavica, Resuscitation, Circulation *and *Critical Care Medicine*. He has published approximately 400 scientific contributions.
